# Photodynamic Therapy for Treatment of Disease in Children—A Review of the Literature

**DOI:** 10.3390/children9050695

**Published:** 2022-05-10

**Authors:** Anna Mazur, Katarzyna Koziorowska, Klaudia Dynarowicz, David Aebisher, Dorota Bartusik-Aebisher

**Affiliations:** 1 Students Biochemistry Science Club URCell, Medical College of the University of Rzeszów, 35-959 Rzeszów, Poland; mazuranna09@gmail.com; 2 Students English Division Science Club, Medical College of the University of Rzeszów, 35-959 Rzeszów, Poland; kasiakozio@icloud.com; 3 Centre for Innovative Research in Medical and Natural Sciences, Medical College of the University of Rzeszów, 35-310 Rzeszów, Poland; kdynarowicz@ur.edu.pl; 4 Department of Photomedicine and Physical Chemistry, Medical College of the University of Rzeszów, 35-959 Rzeszów, Poland; daebisher@ur.edu.pl; 5 Department of Biochemistry and General Chemistry, Medical College of the University of Rzeszów, 35-959 Rzeszów, Poland

**Keywords:** photodynamic therapy, treatment, therapy in children

## Abstract

Photodynamic therapy is a mode of treatment whereby local irradiation of an administered photosensitizer with light of a specific wavelength generates cytotoxic reactive oxygen species. Despite the upward trend in the popularity of this method in adults, it is not yet commonly used in the treatment of children. Due to certain limitations, underdeveloped treatment regimens and potential side effects, the use of photodynamic therapy in the pediatric population is still in the initial phases of evaluation in clinical trials. Method: This study is a review of articles in English from the databases PubMed and Web of Science retrieved by applying the search term “photodynamic therapy in children” from 2000–2020. Results: Based on the literature review, we analyze selected pediatric clinical cases in which photodynamic therapy was used for treatment in children. Examples of photodynamic therapy for treatment of dermatological diseases, diseases of the mucosa of the upper respiratory tract, halitosis, eye diseases and brain tumors are described. The paper describes the effectiveness of anti-cancer photodynamic therapy, including its use in antibacterial therapy. Conclusions: The results of the analysis suggest the potential of photodynamic therapy for the treatment of various diseases in children.

## 1. Diseases in Children

Dermatological problems in children are common ailments that may begin in the neonatal period and last for several years. It is estimated that 30% of patients in dermatology clinics are children [1]. Gorlin’s syndrome or basal cell nevus syndrome is an extremely rare autosomal disease with symptoms such as palmar and plantar pitting [2]. Human papillomaviruses are genital viruses that are usually sexually transmitted; however, there are cases when this disease affects pre-school and school-age children who have not had sexual contact. This virus is the main cause of acuminata, but some genotypes unrelated to genital, perinatal, horizontal or sexual transmission pathways have also been found. The causes of such diseases in children are not fully understood [3]. Another dermatological problem in children is flat warts caused by strains of the human papillomavirus. Warts vary in size but are usually not less than 3 mm and are pink or brown in color. They are most often located on the face, arms, backs of hands and knees and may appear as small, flat warts [4]. Respiratory papillomatosis is a chronic and difficult disease that affects children and adults. It is estimated that the incidence of this disease has a downward trend and is referred to as benign laryngeal cancer in children. This disease is characterized by a high recurrence rate, which makes it a significant problem, especially for the youngest patients [5].

Port-wine stains (PWS) are one of the two most common vascular malformations. These are characterized by the presence of capillaries and veins in the dermis that clinically appear as dark red to purple spots on the skin. These symptoms usually darken and get worse without treatment. This disease also affects children for whom the use of appropriate and effective therapy is a priority. Some patients with lesions may require a combination of treatments. It is worth noting that achieving complete removal of PWS is difficult [6]. These and other diseases, both for children and adults, are a global problem. Given the dynamics of changes and the extent of the disease, it is necessary to implement an effective therapeutic method with a minimum number of side effects. In recent years, PDT has been increasingly introduced as a therapeutic treatment that is an extremely effective form of therapy and, above all, relatively safe.

## 2. Photodynamic Therapy (PDT)

**Figure 1:**
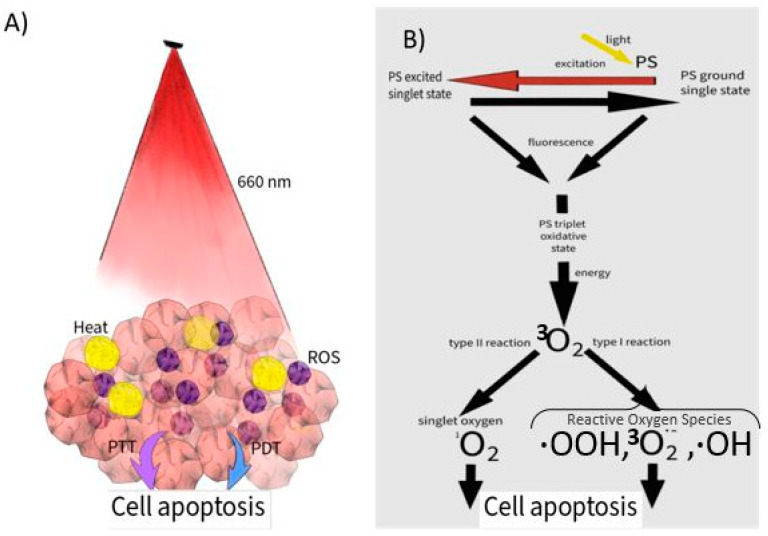
(**A**) Action of PDT in skin tissue. (**B**) The utilization of a photosensitizer (PS) in skin tissue. Clinically approved photosensitizers are used in PDT along with ground-state oxygen (^3^O_2_) and visible light to treat tumors by the local production of cytotoxic singlet oxygen (^1^O_2_). PDT is based on the local application of a photosensitizer that accumulates in pathological tissues. The quality of the therapy depends on the efficiency of the photosensitizer and the place of its deposition in the tissue. The photosensitizer absorbs light of the appropriate wavelength and thus acquires the ability to transfer energy to oxygen molecules in their vicinity, enabling the formation of singlet oxygen that is capable of selectively destroying cancer cells. Highly toxic products are generated during the photodynamic action. The reactive oxygen species formed in this way include singlet oxygen, hydrogen peroxide and hydroxyl radicals. However, for the production of toxic products, it is necessary to use an appropriate photosensitizer.

Photodynamic therapy (PDT) is a modern method that uses light to photoexcite a photosensitizer (PS) to produce reactive oxygen species (ROS), especially singlet oxygen (^1^O_2_) (Figure 1). Photodynamic therapy is used in many fields of medicine, including dermatology, dentistry, gynecology, oncology, but it is in dermatology that it occupies a special place due to the ease of skin irradiation [7,8]. It is also successfully used in inflammatory diseases. Photodynamic therapy is an alternative method of cancer treatment, contributing to improved therapeutic efficacy. Photosensitizers may also act as anti-inflammatory agents, as in PDT it is possible to modulate the expression of inflammatory markers.

In addition to the direct cytotoxic effect leading to cell apoptosis, there are other mechanisms of destruction, such as occlusion of blood vessels and lymphatic and cytokine release. Photosensitizers used in the PDT method accumulate in diseased tissue and are characterized by a high efficiency of conversion to the triplet state and by high light absorption coefficients in the red range or near-infrared light and must not be toxic. Red light with a wavelength of 660 nm is often used for therapeutic irradiation [9,10,11,12]. Photodynamic therapy often requires high-precision light-guidance and dosimetry technology when tumors are located close to important anatomical sites. Areas that are often inaccessible or unsafe to treat with traditional surgical methods include major blood vessels such as the carotid arteries, critical areas of the brain, and parts of the eye. PDT is less invasive than surgery and can be used very precisely.

**Figure 2:**
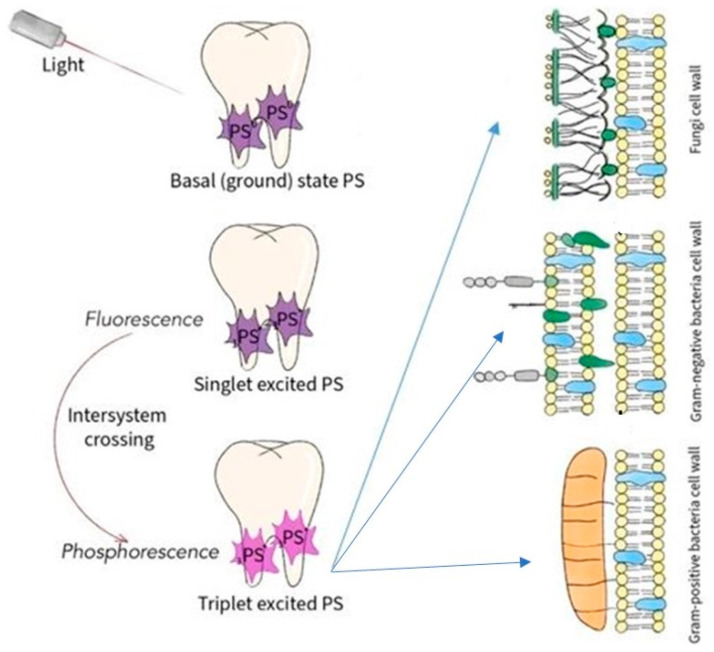
PDT mechanism in dentistry.

Choosing PDT allows patients to avoid complicated surgical procedures. In dentistry, PDT has recently become an experimental approach to assess the effectiveness of dental disease treatment. Antimicrobial PDT (A-PDT) is gaining interest in endodontics to kill microorganisms and other pathogenic bacteria. In dentistry, PDT uses a visible light source of an appropriate wavelength to excite a previously administered photosensitizer, which leads to the elimination of microorganisms (bacteria) without an immune response [13]. Figure 2 shows the mechanisms of PDT in dentists.

After absorption of light, the ground state photosensitizer (PS) is excited to the singlet state (S_1_). After intersystem crossing, the PS enters an excited triplet state. The reaction can occur in two ways: in Type I reactions, photosensitizers can react with molecules by transferring an electron to form radicals; in a Type II reaction, PSs in a triplet state can react directly with oxygen through energy transfer, forming singlet oxygen. Both types of reactions cause cellular cytotoxicity that destroys pathogenic microbes [14]. Photodynamic therapy affects the entire physiology of bacterial and fungal cells, beginning with the destabilization of the cell wall and membrane.

## 3. Materials and Methods

**Figure 3:**
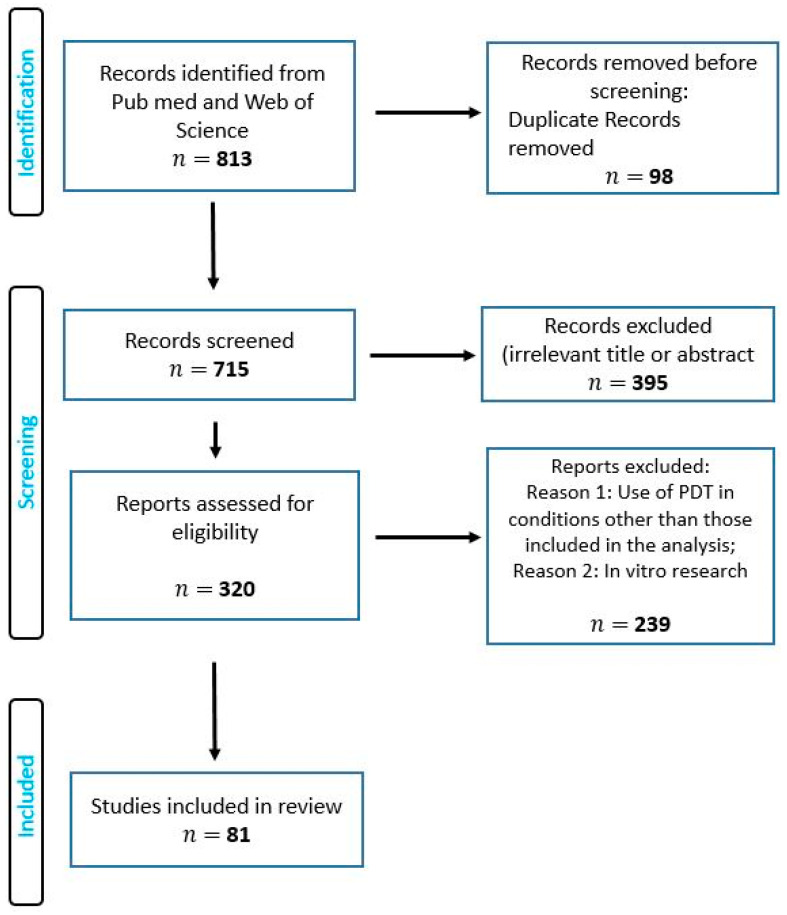
Diagram illustrating the procedure for analyzing the source articles.

For this review, research and review articles from PubMed and Web of Science databases were used. The key words used were: “photodynamic therapy in children” during the years 2000–2020. The total number of papers returned was 813. After dividing the review into topic blocks (the use of PDT in various clinical cases) and analyzing the searched publications, 81 articles were selected. Figure 3 shows a diagram illustrating the procedure for analyzing the source articles.

## 4. Application of Photodynamic Therapy (PDT) for Treatment in Children

As a specialized therapeutic tool, PDT is used in the treatment of bacterial, viral and fungal diseases, in the treatment of cancer, skin diseases, dentistry and ophthalmology [15,16,17]. The first clinical trials in the field of PDT were documented 100 years ago [18]. Oscar Raab was the first to describe the phenomenon of fluorescence resulting from the use of dye and exposure to light as a “photodynamic effect” [19]. There is a lot of research into the use of PDT to treat adult disease. On the other hand, the field of PDT in children is less commonly reported. For this reason, there is much less material on the use of PDT in children compared to clinical trials for adults [20,21,22,23,24]. This review characterizes the research conducted with regard to the use of PDT in the treatment of various types of pediatric disease.

### 4.1. PDT in Dermatological Diseases

Girard et al. used PDT to treat Gorlin’s syndrome and a group of children was included among the qualified patients. It has been shown that the effectiveness of PDT treatment of superficial lesions with the use of 5-aminolevulinic acid (5-ALA) ranges from 78% to 100%. No signs of toxicity were reported in the pediatric study [24].

An article by Lee et al. presented a history of daylight PDT and stated that it may be a less painful, more comfortable and effective treatment alternative. The first clinical applications of PDT in daylight skin cancer treatment began in Copenhagen in 2008 [25].

The first article on daylight PDT was published in 2009. It was a description of PDT using 5-ALA or its methyl ester (MAL) [25], using sunlight as the irradiation source.

There is a report of a 12-year-old boy diagnosed with Bowen’s disease that qualified him for PDT. Methyl aminolevulinate PS and red light were applied. After two PDT sessions, which were carried out intermittently for 3 weeks, the lesion was completely removed. Nine months after the procedure, no recurrences were observed [26].

A study was described by Xu et al. in a group of 12 children with genital warts. Children underwent PDT therapy with the use of a 5-ALA photosensitizer. The light source used was red light with a wavelength of 635 nm for 20 min. Positive changes were observed after the application of PDT therapy. Additionally, no recurrence of the disease was observed. The authors showed that PDT is highly effective in perianal and intra-anal lesions with minimal side effects [27,28]. Plane facial warts (FFW) is a superficial viral skin disease that occurs extremely frequently in childhood. A study assessed the effectiveness of conventional PDT (C-PDT) therapy compared with daylight photodynamic therapy (DL-PDT) using 10% aminolevulinic acid (ALA). To test and evaluate the efficacy and safety of C-PDT versus daylight (DL)-PDT using 10% 5-ALA in the treatment of refractory FFW in children, thirty patients < 18 years of age with facial FFW were randomized into two groups: group A (15 patients) was treated with C-PDT and group B (15 patients) was treated with DL-PDT. The patients were treated three times at 1-month intervals. Response was assessed at weeks 4, 8, 12 and 24 (T4, T8, T12, T24) and rated excellent (75–100% reduction in total warts), very good (74–50% reduction), good (49–25% decrease) or weak (<25% decrease or no response). Each adverse event occurring during or after ALA administration, as well as pain intensity, was recorded at each visit. DL-PDT in combination with 5-ALA was shown to be a safer and more effective method. Compared to C-PDT, it was better tolerated, almost painless and time-saving [4].

Another study described a 6-year-old girl with a year’s history of multiple warts on her foot. The physical examination revealed a subungual papilla and numerous hyperkeratotic plaques on the dorsum of the left foot. The changes did not respond to the earlier cryotherapy performed 3 months earlier; therefore, the conventional PDT method was implemented. After 6 weeks, the lesions were removed with no signs of side effects. Photodynamic therapy may be an alternative therapeutic option for warts, especially in patients refractory to routine treatment. Photodynamic therapy has been shown to kill a variety of microbial cells, including bacteria, fungi and viruses. In the case of warts, it promotes their regression through two mechanisms: generation of cytotoxic radicals that destroy keratinocytes through apoptosis, and stimulation of specific immune responses, releasing various cytokines (interleukin-1β, interleukin-2 and tumor necrosis factor-α). In this study, the warts were removed after just two sessions with minimal side effects and no recurrence. Photodynamic therapy is a relatively new therapy for treating papillary lesions in children. It is especially useful when other treatment methods have failed or showed little improvement, or in those in whom invasive procedures should be avoided [28,29].

Ding et al. described six patients diagnosed with squamous cell warts, mainly on the face, who were treated with PDT with 5-aminolevulinic acid (5-ALA). As a result of the applied therapy, the lesions were completely removed in all six patients after one month of treatment. The results of this study suggest that ALA-PDT may be an effective method for treating squamous nipple cells, especially in cases resistant to other treatments [30]. Chen et al. presented a description of a 9-year-old girl with papillary lesions in the vulva area. Photodynamic therapy was introduced into the therapeutic process. An hour after PDT, the desmosomic junction between the cells appeared visibly fragmented, cell organelles were severely damaged and cytoplasm density became sparse. Four hours after PDT, cells showed remarkable vacuolar degeneration and necrosis. One week after PDT, the papillary lesion was completely removed and the urethral opening was clean. Electron microscopy results showed that PDT mainly damages acanthocytes and coilocytes in virus-infected tissue. As a result of clinical trials, the authors concluded that PDT can be considered an alternative form of therapy in the treatment of papillary lesions [31].

Another condition where PDT has been used is flat-faced warts, which are one of the most common contagious skin conditions. Borgia et al. described the case of an 8-year-old girl treated with PDT who developed clusters on her eyelids, nose and cheeks. For this purpose, the topical t5-ALA cream was used. The light source used was sunlight and not an external laser light source [32]. The method was used twice, with an interval of 1 month between treatments. Six weeks after the last treatment, the scars had completely disappeared without recurrence [32].

Although PDT hemoporfin is a promising treatment for port-wine stains, its effectiveness in children has not been sufficiently evaluated. The authors recommended the introduction of PDT to clinical trials [33]. Hemoporfin-mediated PDT (HMME-PDT) is a vascular-targeted treatment for port-wine (PWS) stains. However, the effectiveness of this varies and is unpredictable. Chun-Hua et al. reviewed the medical records of children with port-wine stains who received PDT. The photosensitizer was hemoporfin (hematoporphyrin monomethyl ether). Hemoporfin PDT uses a green laser light with a wavelength of 532 nm that is applied for approximately 20–25 min [29].

Stain disappearance and adverse events were reported. Ninety five percent of the patients showed an effective response. The patient response to PDT showed a cumulative effect of the treatment session. No photosensitivity or systemic side effects have been observed. Transient local side effects, including edema, purpura, crusts and pigmentation, resolved without treatment. Only 2% of the children had permanent scarring, possibly from scratching the cuticles. This review concluded that hemoporfin photodynamic therapy is well tolerated and effective in pediatric Chinese patients with port-wine stains.

Huang et al. assessed the efficacy of the influencing factors associated with HMME-PDT and provided an appropriate method of predicting efficacy. The study involved 212 patients (93 males) with a mean age of 13.01 ± 12.67 years. The number of PDT treatments is related to efficacy, and patients who received more than three sessions had a better response than those who received fewer than three sessions (*p* = 0.003). Overall, HMME-PDT is an effective treatment for PWS [33].

Li et al. presented observations of 16 PWS patients who received HMME-PDT. As a result, PWS were partially removed. Due to the limited penetration of light into the skin, it is difficult to remove lesions located deeper and with larger diameters [34].

Wang et al. analyzed four patients with PWS of the limbs treated with HMME-PDT. Patients were exposed to green LED light with a wavelength of 532 nm. Three out of four patients were considered cured, and efficacy was estimated to be 75%. Treatment of patients with PWS was highly effective and safe, with no apparent side effects or relapses [35].

Xiao et al. assessed the results and complications of PDT therapy in patients with port-wine lesions. They conducted a total of 3,066 therapy sessions. More than 5% of patients had a total clearance, while 70% of patients had a clearance >25%. Over a quarter of patients (29.8%) experienced removal of over 50% of PWS. Patients responded well and were satisfied with vascular-targeted PDT. The research team admitted that patients with vascular lesions can be treated with an alternative PDT method to obtain a significant therapeutic effect, even if they have minor complications [36].

Yuan et al. and Gracia-Cazaña et al. reviewed PDT clinical trials in patients with port-wine stains in China. The therapy used a laser with a wavelength of 585 nm and a hematoporphyrin photosensitizer. A group of 210 children aged 3 to 10 years with pink lesions in the cheek area were examined [37,38].

Cai et al. presented the case of a 12-year-old patient who was diagnosed with parchment skin and then underwent PDT therapy. After the treatment, most of the skin lesions disappeared within 2 months of the treatment. After the procedure, most of the skin lesions disappeared within 2 months of the procedure, thus confirming the effectiveness of PDT in the treatment of dermatological lesions and parchment skin [39].

### 4.2. Photodynamic Therapy (PDT) in Pediatric Dentistry

Another example of the use of PDT in the treatment of pediatric patients is dentistry. PDT uses an activated dye exposed to light of a specific wavelength in the presence of oxygen to produce reactive oxygen species that damage and destroy microorganisms.

#### 4.2.1. Biofilm

The aim of the research by Alsaif et al. was to determine the most effective bactericidal incubation and irradiation times for erythrosine-based PDT on in vivo-formed plaque biofilms. As a result of the therapy, the percentage of the total number of bacteria decreased (by about 95%). The authors concluded that improving the clinical utility of PDT by shortening the total treatment time appears to be promising and effective in killing platelet biofilms in vivo [40,41,42]. Bargrizan et al. showed that A-PDT kills oral bacteria in plankton culture, plaque and biofilm. This study assessed antimicrobial activity [43]. Rosa et al. assessed the effect of A-PDT as an adjuvant treatment, taking into account the clinical immunoregulatory and microbiological parameters in patients with braces. Characterization was performed on 34 patients of both sexes who had used an appliance for more than 12 months and who developed gingivitis. The photosensitizer was methylene blue, and the light source was a red laser diode with a wavelength of 660 nm. As a result of the therapy, the biofilm was largely removed, reducing the degree of tissue damage. A-PDT is an example of an innovative therapy that ensures uninterrupted orthodontic treatment and thus maintains the patients’ quality of life at a good level [44]. Luke-Marshall et al. also showed that antibacterial PDT, this time with the use of chlorin, has a significant bactericidal effect on biofilms, which makes it a supportive and effective method of treating acute and recurrent diseases related to orthodontics [45]. Okamoto et al. assessed the A-PDT method as an effective form of elimination of microorganisms from the inside of the canal system. The photosensitizer used was methylene blue and the light source was a 660 nm laser. The experiment resulted in a complete (100% efficiency) reduction of bacteria in the root canals [46].

In turn, the aim of the work of Pinheiro et al. was the evaluation of PDT therapy in deciduous teeth with pulp necrosis through the full quantification of live bacteria. Photodynamic therapy was performed with a diode after administration of toluidine blue. As a result of A-PDT, there were reductions in the numbers of microorganisms, with an efficiency of 98.37%. The study concluded that A-PDT is a therapy supporting the reduction of microorganismal populations in deciduous teeth with pulp necrosis [47].

In the case of the Malik and Alkadhi studies, the effectiveness of PDT therapy against oral yeast in children with gingivitis undergoing permanent orthodontic therapy was assessed. The research group consisted of children who underwent orthodontic treatment at the mean age of 16. After the treatment, the numbers of yeasts in oral cavities were significantly lower than before the treatment [48].

The aim of the research conducted by Ri-beiro da Silva et al. was to evaluate the effect of PDT on the treatment of stomatitis in children [49]. A group of 29 patients aged 10 months to 18 years was divided into two groups. One group underwent photodynamic therapy (photosensitizer: 0.01% methylene blue; light source: red laser, wavelength 660 nm). Group II underwent low-level laser therapy. There was a significant reduction in pain in each group. Preservation of deciduous teeth with pulp changes caused by caries or trauma is the main therapeutic challenge in pediatric dentistry. Antibacterial Photodynamic Therapy (A-PDT) is a new and promising complementary therapy in endodontic treatment to eliminate persistent microbes after chemo-mechanical preparation. In her article, De Sant’Anna presented the case of a 5-year-old boy diagnosed with type I diabetes who, due to an injury, required pulp treatment in the primary left medial incisor of the jaw. The proposed treatment included the use of PDT for the decontamination of root canals with the use of a methylene blue dye at a concentration of 50 μg/mL. Modern laser technology and related therapies have brought significant benefits beyond conventional endodontic therapy [50]. The proposed endodontic treatment consisted of PDT-related chemo-mechanical treatment in root canal decontamination with 50 μg/mL methylene blue for 3–5 min [44]. Da Mota et al. assessed the efficacy of PDT in clinical trials of endodontic treatment of primary teeth [50]. The authors included children aged 3 to 6 years, who were divided into a research group (treated with PDT) and a control group (with conventional treatment). The children struggled with caries or other traumas accompanied by involvement of the pulp. The photosensitizer used was methylene blue with a concentration of 0.005%. Root canals were irradiated with a laser with a length of 660 nm, an energy of 4 J and an average power of 100 mW for 40 s. In turn, in conventional treatment, surgical chemical preparation was performed. The experiment confirmed that A-PDT is a painless, easy-to-use method that does not lead to microbial resistance and can help achieve effective endodontic treatment of teeth by eliminating the pain that children may experience during re-treatment, as well as preventing premature tooth loss.

#### 4.2.2. Caries

Fekrazad et al. conducted research on the reduction of pathogenic microorganisms in children with severe caries. The research group was a group of twenty-two children with severe caries in early childhood, aged 3–6. The children were treated with toluidine blue for 1 min and irradiated with a light-emitting diode for 150 s. Saliva samples from each treated child were collected in three stages: before the examination, 1 h after the procedure and 7 days after the procedure. The number of *Streptococcus mutants* was determined using Dentocult SM Strip mutans to assess the density of bacterial colonies. For this purpose, the software Statistical Package for Social Sciences 22.0 (SPSS Inc, Chicago, IL, USA, 111) was used to perform the Wilcoxon test. The significance level (*p*-value) was 0.001. The mean values of the numbers of *Streptococcus mutans* at each measurement step were as follows. At baseline (before treatment), the mean value of *Streptococcus mutans* was 2.32 ± 0.65. One hour after surgery, the number of *Streptococcus mutans* was 1.64 ± 1.09. However, a measurement made 7 days after treatment showed that the number of bacteria had already increased to 2.23 ± 0.68. After the experiment, the authors concluded that the number of *Streptococcus mutans* in saliva decreased significantly after 1 h. However, 7 days after treatment, Streptococcus mutans levels returned to baseline levels. Therefore, A-PDT seems to be efficient to reduce *Streptococcus mutans* immediately after treatment in children with severe caries in early childhood [51]. Similar conclusions were presented by Alves et al., who confirmed the efficacy of PDT against cariogenic microorganisms after selective caries removal without damaging the composites of teeth [52]. A-PDT is an alternative method of supporting root canal decontamination [53] and is an alternative form of caries treatment. The aim of the work by Méndez et al. and Costa-Santos et al. was the assessment of the effect of A-PDT on the viability of microorganisms, biofilm and the production of lactic acid in dentin carious microvilli [53,54]. As a result of the experiment, the total number of microorganisms was reduced. The amount of lactic acid produced was lower than in the group where A-PDT was not used. The experiment confirmed the effectiveness of A-PDT in controlling the viability and acidity of the microvilli of dentin caries [54]. The aim of the studies by Potapczuk et al. was the evaluation of the effectiveness of the treatment of dental caries in children with PDT. The research group consisted of 35 children aged 12–15 years. The polymerase chain reaction method was used to determine the efficacy of A-PDT in the treatment of dentin caries. As a result of the therapy, the number of bacterial cells, such as Enterococcus faecalis, Veilonella and Candida albicans, decreased. After the experiment, it was found that PDT in the treatment of dentin caries is a highly effective and pathogenetically proven method of treatment, ensuring a significant reduction of facultative and obligatory types of carious microorganisms [55]. Carvalho et al. described the testing of chemical–mechanical and antimicrobial methods of A-PDT photodynamic therapy in combination with deep caries in permanent pediatric molars. Due to the specificity of early erupting teeth as the first permanent lower molars, techniques that provide less invasive tissue removal in order to maintain healthy parts of teeth, such as the pulp, are desirable. In addition, they provide comfort, especially for children, less noise and vibration, which are important in the treatment of deep caries. The paper describes the effect of A-PDT in a 9-year-old patient with deep caries of the first right mandibular molar. Six months after the A-PDT treatment with Rose Bengal, no traces of caries were found, which confirmed the effectiveness of the technique used. A case–control study of A-PDT with a toluidine blue diode laser on mutant salivary streptococci in children aged 5–6 years with severe caries in early childhood was performed on 56 children. Bacterial counts decreased significantly on days 1 and 3, and between weeks 1 and 2 after the second intervention [56,57,58].

### 4.3. PDT in Diseases of the Mucosa of the Upper Respiratory Tract

The PDT method is also used in the case of recurrent respiratory papillomatosis. This disease is a mild disease of the upper respiratory mucosa. It is characterized by frequent relapses and rapid growth of papilloma, making it a potentially life-threatening disease. A large percentage of patients suffering from this disease require multiple surgical procedures characterized by a high degree of invasiveness. One form of supportive care is PDT. Lieder et al. analyzed a group of children and adults diagnosed with respiratory papillomatosis. The authors concluded that PDT as a standalone therapy and in combination with surgical treatment brings therapeutic benefits [59]. Another example of the use of PDT in respiratory papillomatosis is a clinical study by Shikowitz et al. The research group consisted of 23 patients (children and adults diagnosed with recurrent respiratory papillomatosis [60]. The photosensitizer used was meso-tetra (hydroxyphenyl) chlorin (m-THPC). As a result of the therapy, the severity of the disease decreased and immune response improved [61]. A few years earlier, in 1998, Shikowitz et al. also conducted research on PDT in respiratory papillomatosis. As a result of the therapy, a decrease in the growth rate of papilloma was also observed [61].

### 4.4. PDT for the Treatment of Halitosis

Halitosis is chronically bad breath that does not go away after you brush your teeth. It is quite common and it is difficult to deal with. One of the methods of therapy for children and adolescents is PDT. Costa da Moto et al. undertook studies to evaluate the efficacy of A-PDT. The study showed that applying PDT to the back of the tongue had an immediate effect. There was a reduction in mouth malodor by reducing the concentration of hydrogen sulfuric acid [62]. Alshahrani et al. evaluated the effectiveness of PDT therapy in the treatment of halitosis in a group of adolescent patients who received orthodontic treatment. The research group consisted of 45 patients. The mean age of the patients was 14–15 years. As a result of the therapy, there was an immediate reduction in the concentration of hydrogen sulfide and a reduction of pathogens in the oral cavity [63].

### 4.5. PDT in Eye Disease

Choroidal neovascularization is the growth of small vessels originating from the choroid capillaries, which, through Bruch’s membrane, enter the space under the retinal pigment epithelium, as well as retinal pigment epithelial cells and photoreceptors. Pathological vessels are defective, less durable, more fragile and have a tortuous course. Leakage of newly formed vessels results in leakage of fluid or blood, which accumulates in the subretinal space, causing hemorrhagic detachment of the retinal pigment epithelium. This leads to the formation of a discoid, fibrous vascular scar. Ozdek et al. undertook research in the treatment of choroidal neovascularization. They analyzed a group of four children [64]. The results of clinical trials were analyzed using fluorescein angiography and optical coherence tomography. All patients responded well to PDT. The effectiveness of improving visual acuity was 80%. The increase or stabilization of visual acuity was maintained over a mean follow-up of 25 months. The aim of research by Lipski et al. was to determine the therapeutic potential of PDT using verteporfin in patients with choroidal neovascularization [65]. The obtained results indicated a good efficacy and tolerability of PDT in the group of patients with visual disturbances unrelated to active uveitis. Photodynamic therapy in young patients remains a valuable treatment with a good benefit–risk profile over the long term. Süsskind et al. started treatment of the choroidal vessel with PDT [66]. The aim of the study was to evaluate the effectiveness of PDT in the treatment of exudative limited choroidal hemangioma. Average visual acuity improved after treatment. Exudate retention occurred in several patients. In turn, the mean thickness of the fovea located by optical computed tomography was reduced. Photodynamic therapy has been shown to be an effective, safe procedure in the treatment of exudative choroidal hemangioma. The number of side effects was small. Similar observations were proposed by Yıldırım et al. They analyzed a 10-year-old girl who had lost sight in her right eye. Photodynamic therapy with verteporfin was used in the therapy. After four months of therapy, visual acuity increased to 80%. As a result of PDT, the subretinal fluid completely resolved [67]. After one year of follow-up, visual acuity and fundus were stable with no recurrence. In turn, Giansanti et al. included five patients aged 7–15 years who were treated with PDT in a study. As a result of PDT, infiltration decreased, and there were atrophic changes in the retinal pigment epithelium. Visual acuity was stable [68]. Sturge–Weber syndrome, also known as facial–cerebral nomenclature, is a group of neurocutaneous developmental disorders defined by phacomatosis. Nugent et al. treated a 6-year-old girl with Sturge–Weber syndrome with PDT. As a result of the applied PDT, retinal detachment completely resolved within 3 months after treatment [69]. Thus, the effectiveness of PDT was also confirmed in a case of hemangioma in a child with Sturge–Weber syndrome. Mauget-Faÿsse et al. evaluated the efficacy and safety of PDT in combination with verteporfin in children and young people with subfoveal choroidal neovascularization [70]. As a result of PDT, visual acuity increased, a vascular anastomosis developed and no significant side effects were observed. Farah et al. evaluated the role of PDT using verteporfin in the treatment of subfoveal choroidal neovascularization in Vogt–Koyanagi–Harada syndrome. A 9-year-old patient was analyzed in whom complete regression of the lesion was observed within 1 week after treatment [71].

### 4.6. PDT in Brain Tumors

Brain tumors in children and neoplasms of the nervous system occurring in childhood differ significantly from neoplasms found in adults. The observed differences concern mainly the location of tumors and their histopathology. The specificity of childhood also affects their course and the possibility of treating tumors, both surgically and with chemotheraputic methods. The above differences are so significant that they have given reason to distinguish specialist centers of pediatric neurosurgery and neuro-oncology, which specialize in and treat brain tumors in children. The choice of the form of treatment should be carefully selected as not every form of treatment used in adult patients can be used in children. An example of a method used in pediatric clinical trials is PDT, in which both in vitro and in vivo studies are being carried out. Schwake et al. conducted in vitro studies on PDT in pediatric brain tumor cells. To this end, they used four different central nervous system tumor cell lines. The light source used was a diode with a wavelength of 635 nm. The exposure time was 250 s. The photosensitizers used were 5-ALA and protoporphyrin. The experiment showed that PDT resulted in significant death of malignant tumor cells [72]. In studies by Schmidt et al., twenty patients with recurrent malignant brain tumors were subjected to PDT [73]. The photosensitizer used was a porphyrin, while the light source was a light-emitting diode (LED). All treated patients showed a tumor response, as analyzed by MRI. Lou et al. analyzed interstitial photodynamic therapy, which consists of treating internal tumors with light provided by fibers inserted percutaneously. Clinical trials have confirmed the hypothesis that interstitial photodynamic therapy provides a valuable alleviation treatment with few complications and occasional long-term experiences in terminal advanced head and neck cancer. This is a treatment option worth adding to those available for integrated head and neck cancer syndromes [74]. PDT has the potential to become an effective therapy in pediatrics and is applied in the treatment of osteosarcoma [75], dermatologic conditions [76], evaluations of immunomodulatory activity [77], central serous chorioretinopathy [78], hepatoblastoma [79] and bulimia nervosa [80] in adults. Photodynamic therapy is an innovative method of treating cancer with the potential to supplement chemotherapy and radiation therapy. The advantage of this method is the low number of side effects, which are particularly troublesome in pediatric patients. During illness, children undergoing traditional chemotherapy complain not only of pain but also nausea and vomiting. Photodynamic therapy used in pediatric patients is increasingly being used worldwide with satisfactory results. Increasing research on the application of PDT in neoplastic diseases in children presents an opportunity to develop an effective method of supporting treatment and constituting the basis for non-surgical, less harmful treatment in which chemotherapy or radiation therapy can be limited. PDT offers perspectives on increasing the survival of cancer patients who receive this method of treatment. Table 1 summarizes the information from the review of the articles.

## 5. Limitations

One of the limitations of the use of PDT in children is the selection and use of non-toxic photosensitizers. Additionally, the amount of photosensitizer administered must be kept to a minimum (definitely lower than in adults) for efficient elimination. Due to the fact that the photosensitizer is administered in advance of exposure to light, the patient may react differently to the relatively long waiting period, which may vary from several minutes to several days. In deeply located lesions, the photosensitizer is administered intravenously, which can be very uncomfortable, especially in young patients [21].

## 6. Conclusions

This report highlights PDT as a modern method of treatment for a variety of diseases in children. In this technique, after systemic administration of a photosensitizing agent (PS), local necrosis of neoplastic cells, inflammatory lesions or cells of microorganisms, such as bacteria, viruses and fungi, is induced by the generation of cytotoxic ROS. The photosensitizer is one of the most important factors responsible for the successful performance of light therapy. Photodynamic therapy is a new method of fighting malignant neoplasms and bacteria. The use of this therapy allows a reduction in the number of surgical procedures and often successfully completes the treatment process. In all the analyzed clinical cases in children (in dermatological and dental diseases, in diseases of the mucosa of the upper respiratory tract, in eye diseases and in brain tumors) in which PDT was used, treatment results were improved. Regarding dermatological changes, the effectiveness of PDT ranged from 78–100%, which confirms its importance in the treatment of dermatological diseases in children. A small percentage of side effects occurring during the PDT procedure confirm the non-invasiveness and safety of this method. Similar conclusions were drawn when analyzing the use of PDT in treating dental diseases and halitosis. The diseases most often treated with PDT are caries and stomatitis and it is also used in the treatment of root canal decontamination. The effectiveness of the therapy was almost 100%. In turn, in the treatment of respiratory papillomatosis, PDT is an adjunctive therapy that reduces the rate of disease development. In the treatment of eye diseases, such as choroidal neovascularization, choroidal hemangioma and Sturge–Weber syndrome, the efficacy of PDT was also high. Its average value was 80%. The last group of diseases mentioned in this review were clinical cases of brain tumors in which PDT was one of the therapeutic tools employed. In all cases, PDT resulted in a reduction in the number of neoplastic cells.

## Figures and Tables

**Table 1:** Effectiveness of PDT therapy.

Clinical Case	Photosensitizers	Light Source	Efficacy of PDT	References
**Gorlin’s syndrome**	MAL cream (Metvixia methyl aminolevulinate cream 16.8%)	Red light (Aktilite lamp; Photocure-Galderma, CL128 LED) 635° nm	78–100%	[24]
**Bowen’s disease**	Methyl aminolevulinate	Red light	100%	[26]
**Genital warts**	5-aminolevulinic acid	Red light at 635 nm (LD600 C. Wuhan Yage Optic and Electronic Technique Co., Ltd., Wuhan, China)	High efficacy	[27]
**Genital warts**	5-aminolevulinic acid	Red light at 630 nm (S630, AlphaStrumenti, Milan, Italy, fluence: 75 J/cm^2^)	High efficacy	[28]
**Multiple warts**	Hematoporphyrin monomethyl ether	532 nm green LED light (LED Therapeutic Machine, LED-IE, Wuhan YaGe Optic and Electronic Technique Co., Ltd., Wuhan, Hubei, China) power density of 80–85 mW/cm^2^, energy density of 96–115 J/cm^2^	80–100%	[29]
**Squamous cell warts**	5-aminolevulinic acid	LED red light source (633 ± 10 nm narrow band, LED-IB) initial irradiance: 80 mW/cm^2^	100%	[30]
**Papillary lesions in the vulva area**	5-aminolevulinic acid	630° nm wavelength He-Ne laser light	90–100%	[31]
**Port-wine stains**	Hematoporphyrin monomethyl ether	532 nm LED light (Wuhan YaGe Laser Engineering), power density ranging 80–100 mW/cm^2^.	High efficacy	[33]
**Port-wine stains**	Hematoporphyrin monomethyl ether	532 nm green light (produced by Wuhan Accord)	75%	[35]
**Port-wine lesions**	Hematoporphyrin monomethyl ether	IPCu-10 copper vapor laser; Huihong Electronic Science Technology, Ningbo, Zhe Jiang Province, China	Over 50%	[36]
**Port-wine stains**	Hematoporphyrin monomethyl ether	Copper vapour laser (510.6 and 578.2 nm)	95.5%	[37]
**Biofilm reduction**	Toluidine blue O (TBO) Methylene blue	Red diode laser (633 nm, 20 mW, 6 J/cm^2^)	95%	[43]
**Oral problems**	Methylene blue	Diode laser (633 nm, 20 mW, 6 J/cm^2^)	High efficacy	[55]

## Data Availability

The data presented in this study is available by request from the corresponding author.

## References

[1] Sethuraman G., Bhari N. (2014). Common skin problems in children. Indian J. Pediatr..

[2] Bresler S.C., Padwa B.L., Granter S.R. (2016). Nevoid basal cell carcinoma syndrome (Gorlin Syndrome). Head Neck Pathol..

[3] Jayasinghe Y., Garland S.M. (2006). Genital warts in children: What do they mean. Arch. Dis. Child..

[4] Borgia F., Giuffrida R., Coppola M., Princiotta R., Vaccaro M., Guarneri F., Cannavò S.P. (2020). Efficacy and safety of conventional versus daylight photodynamic therapy in children affected by multiple facial flat warts. Photodiagn. Photodyn. Ther..

[5] Derkay C.S., Bluher A.E. (2019). Update on recurrent respiratory papillomatosis. Otolaryngol. Clin. N. Am..

[6] Updyke K.M., Khachemoune A. (2017). Port-wine stains: A focused review on their management. J. Drugs Dermatol..

[7] Acedo P., Stockert J.C., Cañete M., Villanueva A. (2014). Two combined photosensitizers: A goal for more effective photodynamic therapy of cancer. Cell Death Dis..

[8] Dos Santos A.F., Terra L.F., Wailemann R.A., Oliveira T.C., Gomes V.M., Mineiro M.F., Meotti F.C., Bruni-Cardoso A., Baptista M.S., Labriola L. (2017). Methylene blue photodynamic therapy induces selective and massive cell death in human breast cancer cells. BMC Cancer.

[9] Agostinis P., Berg K., Cengel K.A., Foster T.H., Girotti A.W., Gollnick S.O., Hahn S.M., Hamblin M.R., Juzeniene A., Kessel D. (2011). Photodynamic therapy of cancer: An update. CA Cancer J. Clin..

[10] Dougherty T.J., Marcus S.L. (1992). Photodynamic therapy. Eur. J. Cancer.

[11] Celli J.P., Spring B.Q., Rizvi I., Evans C.L., Samkoe K.S., Verma S., Pogue B.W., Hasan T. (2010). Imaging and photodynamic therapy: Mechanisms, monitoring, and optimization. Chem. Rev..

[12] Juzeniene A., Peng Q., Moan J. (2010). Milestones in the development of photodynamic therapy and fluorescence diagnosis. Photochem. Photobiol. Sci..

[13] Abrahamse H., Hamblin M.R. (2016). New photosensitizers for photodynamic therapy. Biochem. J..

[14] Diogo P., Faustino M.A.F., Neves M.G.P.M.S., Palma P.J., Baptista I., Gonçalves T., Santos J.M. (2019). An insight into advanced approaches for photosensitizer optimization in endodontics—A critical review. J. Funct. Biomater..

[15] Kwiatkowski S., Knap B., Przystupski D., Saczko J., Kędzierska E., Knap-Czop K., Kotlinska J., Michel O., Kotowski K., Kulbacka J. (2018). Photodynamic therapy-mechanisms, photosensitizers and combinations. Biomed. Pharmacother..

[16] Dynarowicz K. (2021). Naturally occurring photosensitizers and photodynamic therapy: Laser or sun?. Eur. J. Clin. Exp. Med..

[17] Krupka M., Bożek A., Bartusik-Aebisher D., Cieślar G., Kawczyk-Krupka A. (2021). Photodynamic therapy for the treatment of infected leg ulcers- a pilot study. Antibiotis.

[18] Daniell M.D., Hill J.S. (1991). A history of photodynamic therapy. Aust. N. Z. J. Surg..

[19] Kick G., Messer G., Plewig G. (1996). Historical development of photodynamic therapy. Hautarzt.

[20] Kumar N., Warren C.B. (2017). Photodynamic therapy for dermatologic conditions in the pediatric population: A literature review. Photodermatol. Photoimmunol. Photomed..

[21] Gunaydin G., Gedik M.E., Ayan S. (2021). Photodynamic Therapy—Current Limitations and Novel Approaches. Front. Chem..

[22] Sandberg C., Stenquist B., Rosdahl I., Ros A.M., Synnerstad I., Karlsson M., Gudmundson F., Ericson M.B., Larko O., Wennberg A.-M. (2006). Important factors for pain during photodynamic therapy for actinic keratosis. Acta Derm. Venereol..

[23] Smits T., Moor A.C. (2009). New aspects in photodynamic therapy of actinic keratoses. J. Photochem. Photobiol. B.

[24] Girard C., Debu A., Bessis D., Blatiére V., Dereure O., Guillot B. (2013). Treatment of Gorlin syndrome (nevoid basal cell carcinoma syndrome) with methylaminolevulinate photodynamic therapy in seven patients, including two children: Interest of tumescent anesthesia for pain control in children. J. Eur. Acad. Dermatol. Venereol..

[25] Lee C.N., Hsu R., Chen H., Wong T.W. (2020). Daylight Photodynamic Therapy: An Update. Molecules.

[26] Hyun D.J., Seo S.R., Kim D.H., Yoon M.S., Lee H.J. (2016). Periungual Bowen’s disease in a 12-year-old boy treated with photodynamic therapy. Pediatr. Dermatol..

[27] Xu M., Lin N., Li J., Jiang L., Zeng K. (2018). Photodynamic therapy as an alternative therapeutic option for pediatric candyloma acuminate: A case series. Photodiagn. Photodyn. Ther..

[28] Borgia F., Giuffrida R., Coppola M., Cannavò S.P. (2020). Successful photodynamic therapy in a pediatric patient with difficult warts. Dermatol. Ther..

[29] Chun-Hua T., Li-Qiang G., Hua W., Jian Z., Si-Li N., Li L., Yi W., Can L., Xiao-Yan L., Guang-Hui W. (2021). Efficacy and safety of hemoporfin photodynamic therapy for port-wine stains in paediatric patients: A retrospective study of 439 cases at a single centre. Photodiagn. Photodyn. Ther..

[30] Ding A., Li C., Zhang J. (2021). Topical 5-aminolevulinic acid photodynamic therapy in the treatment of verruca plana: Report of 6 cases. Photodiagn. Photodyn. Ther..

[31] Chen M., Xie J., Han J. (2010). Photodynamic therapy of condyloma acuminatum in a child. Pediatr. Dermatol..

[32] Borgia F., Coppola M., Giuffrida R., Cannavò S.P. (2019). Excellent cosmetic result of daylight photodynamic therapy for facial flat warts in a child. Photodiagn. Photodyn. Ther..

[33] Huang Y., Yang J., Sun L., Zhang L., Bi M. (2021). Efficacy of influential factors in hemoporfin-mediated photodynamic therapy for facial port-wine stains. J. Dermatol..

[34] Li Y., Wang X., Liu Y., Tao J. (2020). Dermoscopy predicts outcome in hemoporfin-mediated photodynamic therapy of port-wine stains: A prospective observational study. J. Am. Acad. Dermatol..

[35] Wang S., Lee L.Y., Liu S.X. (2020). Photodynamic therapy for port-wine stains in extremities: Report of 4 cases. Photodiagn. Photodyn. Ther..

[36] Xiao Q., Li Q., Yuan K.H., Cheng B. (2011). Photodynamic therapy of port-wine stains: Long-term efficacy and complication in Chinese patients. J. Dermatol..

[37] Yuan K.H., Li Q., Yu W.L., Zeng D., Zhang C., Huang Z. (2008). Comparison of photodynamic therapy and pulsed dye laser in patients with port wine stain birth marks: A retrospective analysis. Photodiagn. Photodyn. Ther..

[38] Gracia-Cazaña T., Vera-Álvarez J., García-Patos V., Gilaberte Y. (2015). Imiquimod and photodynamic therapy are useful in the treatment of porokeratosis in children with bone marrow transplantation. Pediatr. Dermatol..

[39] Cai H., Yang Q.Q., Ma C., Zou D.X., Wang Y.X., Sun P., Ju A.-Q., Fang F., Gong S., Liu W. (2020). Photodynamic therapy in the treatment of xeroderma pigmentosum: A case report. Photodiagn. Photodyn. Ther..

[40] Alsaif A., Tahmassebi J.F., Wood S.R. (2021). Treatment of dental plaque biofilms using photodynamic therapy: A randomised cotrolled study. Eur. Arch. Paediatr. Dent..

[41] Vieira L.D.S., Paschoal M.A.B., de Barros Motta P., Ferri E.P., Ribeiro C.D.P.V., Dos Santos-Pinto L.A.M., Motta L.J., Goncalves M.L.L., Horliana A.C.R.T., Fernandes K.P.S. (2019). Antimicrobial photodynamic therapy on teeth with molar incisor hypomineralization-controlled clinical trial. Medicine.

[42] De Oliveira F.S., Cruvinel T., Cusicanqui Méndez D.A., Dionísio E.J., Rios D., Machado M.A.A.M. (2018). The in vitro effect of antimicrobial photodynamic therapy on dental microcosm biofilms from partially erupted permanent molars: A pilot study. Photodiagn. Photodyn. Ther..

[43] Bargrizan M., Fekrazad R., Goudarzi N., Goudarzi N. (2019). Effects of antibacterial photodynamic therapy on salivary mutans streptococci in 5-to-6-year-olds with severe early childhood caries. Lasers Med. Sci..

[44] Rosa E.P., Murakami-Malaquias-Silva F., Schalch T.O., Teixeira D.B., Horliana R.F., Tortamano A., Buscariolo I.A., Longo P.L., Negreiros R.M., Bussadori S.K. (2020). Efficacy of photodynamic therapy and periodontal treatment in patients with gingivitis and fixed orthodontic appliances: Protocol of randomized, controlled, double-blind study. Medicine.

[45] Luke-Marshall N.R., Hansen L.A., Shafirstein G., Campagnari A.A. (2020). Antimicrobial photodynamic therapy with chlorin e6 is bactericidal against biofilms of the primary human otopathogens. mSphere.

[46] Okamoto C.B., Motta L.J., Prates R.A., da Mota A.C.C., Gonçalves M.L.L., Gonçalves M.L.L., Horliana A.C.R.T., Ferrari R.A.M., Fernandes K.P.S., Bussadori S.K. (2018). Antimicrobial photodynamic therapy as a co-adjuvant in endodontic treatment of deciduous teeth: Case series. Photochem. Photobiol..

[47] Pinheiro S.L., Schenka A.A., Neto A.A., de Souza C.P., Rodriguez H.M., Ribeiro M.C. (2009). Photodynamic therapy in endodontic treatment of deciduous teeth. Lasers Med. Sci..

[48] Malik N.K.A., Alkadhi O.H. (2020). Effectiveness of mechanical debridement with and without antimicrobial photodynamic therapy against oral yeasts in children with gingivitis undergoing fixed orthodontic therapy. Photodiagn. Photodyn. Ther..

[49] Ribeiro da Silva V.C., da Motta Silveira F.M., Barbosa Monteiro M.G., da Cruz M.M.D., Caldas Júnior A.F., Pina Godoy G. (2018). Photodynamic therapy for treatment of oral mucositis: Pilot study with pediatric patients undergoing chemotherapy. Photodiagn. Photodyn. Ther..

[50] Da Mota A.C., Gonçalves M.L., Bortoletto C., Olivan S.R., Salgueiro M., Godoy C., Altavista O.M., Pinto M.M., Horliana A.C., Motta L.J. (2015). Evaluation of the effectiveness of photodynamic therapy for the endodontic treatment of primary teeth: Study protocol for a randomized controlled clinical trial. Trials.

[51] Fekrazad R., Seraj B., Chiniforush N., Rokouei M., Mousavi N., Ghadimi S. (2017). Effect of antimicrobial photodynamic therapy on the counts of salivary Streptococcus mutans in children with severe early childhood caries. Photodiagn. Photodyn Ther..

[52] Alves L.V.G.L., Curylofo-Zotti F.A., Borsatto M.C., Salvador S.L.S., Valério R.A., Souza-Gabriel A.E., Corona S.A.M. (2019). Influence of antimicrobial photodynamic therapy in carious lesion. Randomized split-mouth clinical trial in primary molars. Photodiagn. Photodyn Ther..

[53] Méndez D.A.C., Gutierrez E., Dionísio E.J., Oliveira T.M., Buzalaf M.A.R., Rios D., Maam M., Cruvinel T. (2018). Effect of methylene blue-mediated antimicrobial photodynamic therapy on dentin caries microcosms. Laser Med. Sci..

[54] Costa-Santos L., Silva-Júnior Z.S., Sfalcin R.A., da Mota A.C.C., Horliana A.C.R.T., Motta L.J., Mesquita-Ferrari R.A., Fernandes K.P.S., Prates R.A., Silva D.F.T. (2019). The effect of antimicrobial photodynamic therapy on infected dentin in primary teeth: A randomized controlled clinical trial protocol. Medicine.

[55] Potapchuk A.M., Almashi V.M., Lomnitsky I.Y., Rusyn V.V., Hegedush V. (2020). The use of photodynamic therapy in the treatment of dental caries in children of contaminated areas of the ecosystem of the upper tysa region. Wiad Lek..

[56] Carvalho L.T., Belém F.V., Gonçalves L.M., Bussadori S.K., Paschoal M.A.B. (2020). Chemo-mechanical and photodynamic approach in A deep dental cavity: A case report. Photodiagn. Photodyn Ther..

[57] De Sant’Anna G. (2014). Photodynamic therapy for the endodontic treatment of a traumatic primary tooth in a diabetic pediatric patient. J. Dent. Res. Dent. Clin. Dent. Prospects.

[58] da Silva Barbosa P., Duarte D.A., Leite M.F., de Sant’ Anna G.R. (2014). Phtodynamic therapy in pediatric dentistry. Case Rep. Dent..

[59] Lieder A., Khan M.K., Lippert B.M. (2014). Photodynamic therapy for recurrent respiratory papillomatosis. Cochrane Database Syst. Rev..

[60] Shikowitz M.J., Abramson A.L., Steinberg B.M., DeVoti J., Bonagura V.R., Mullooly V., Nouri M., Ronn A.M., Inglis A., McClay J. (2005). Clinical trial of photodynamic therapy with meso-tetra (hydroxyphenyl) chlorin for respiratory papillomatosis. Arch. Otolaryngol. Head Neck Surg..

[61] Shikowitz M.J., Abramson A.L., Freeman K., Steinberg B.M., Nouri M. (1998). Efficacy of DHE photodynamic therapy for respiratory papillomatosis: Immediate and long-term results. Laryngoscope.

[62] Da Mota A.C.C., França C.M., Prates R., Deana A.M., Costa Santos L., Garcia R.L., Goncales M.L.L., Ferrari R.A.M., Fernandes K.P.S., Bussadori S.K. (2016). Effect of photodynamic therapy for the treatment of halitosis in adolescents- a controlled, microbiological, clinical trial. J. Biophotonics.

[63] Alshahrani A.A., Alhaizaey A., Kamran M.A., Alshahrani I. (2020). Efficacy of antimicrobial photodynamic therapy against halitosis in adolescent patients undergoing orthodontic treatment. Photodiagn. Photodyn. Ther..

[64] Ozdek S., Ozmen M.C., Tufan H.A., Gurelik G., Hasanreisoglu B. (2012). Photodynamic therapy for best disease complicated by choroidal neovascularization in children. J. Pediatr. Ophthalmol. Strabismus.

[65] Lipski A., Bornfeld N., Jurklies B. (2008). Photodynamic therapy with verteporfin in paediatric and young adult patients: Long-term treatment results of choroidal neovascularisations. Br. J. Ophthalmol..

[66] Süsskind D., Inhoffen W., Gelisken F., Völker M. (2018). Photodynamic therapy with double duration for circumscribed choroidal haemangioma: Functional and anatomical results based on initial parameters. Clin. Exp. Ophthalmol..

[67] Yıldırım C., Çetin E.N., Yayla K., Avunduk A.M., Yaylalı V. (2011). Photodynamic therapy for unilateral idiopathic peripapillary choroidal neovascularization in a child. Int. Ophthalmol..

[68] Giansanti F., Virgili G., Varano M., Tedeschi M., Rapizzi E., Giacomelli G., Menchini U. (2005). Photodynamic therapy for choroidal neovascularization in pediatric patients. Retina.

[69] Nugent R., Lee L., Kwan A. (2015). Photodynamic therapy for diffuse choroidal hemangioma in a child with Sturge-Weber syndrome. J. AAPOS.

[70] Mauget-Faÿsse M., Mimoun G., Ruiz-Moreno J.M., Quaranta-El Maftouhi M., De Laey J.J., Postelmans L., Soubrane G., Defauchy M., Leys A. (2006). Verteporfin photodynamic therapy for choroidal neovascularization associated with toxoplasmic retinochoroiditis. Retina.

[71] Farah M.E., Costa R.A., Muccioli C., Guia T.A., Belfort R. (2002). Photodynamic therapy with verteporfin for subfoveal choroidal neovascularization in Vogt-Koyanagi-Harada syndrome. Am. J. Ophthalmol..

[72] Schwake M., Nemes A., Dandrop J., Schroeteler J., Schipmann S., Senner V., Stummer W., Ewelt C. (2018). In-Vitro use of 5-ALA for photodynamic therapy in pediatric brain tumors. Neurosurgery.

[73] Schmidt M.H., Meyer G.A., Reichert K.W., Cheng J., Krouwer H.G., Ozker K., Whelan H.T. (2004). Evaluation of photodynamic therapy near functional brain tissue in patients with recurrent brain tumors. J. Neurooncol..

[74] Lou P.J., Jäger H.R., Jones L., Theodossy T., Bown S.G., Hopper C. (2004). Interstitial photodynamic therapy as salvage treatment for recurrent head and neck cancer. Br. J. Cancer.

[75] Yu W., Zhu J., Wang Y., Wang J., Fang W., Xia K., Shao J., Wu M., Liu B., Liang C. (2017). A review and outlook in the treatment of osteosarcoma and other deep tumors with photodynamic therapy: From basic to deep. Oncotarget.

[76] Lee Y., Baron E.D. (2011). Photodynamic therapy: Current evidence and applications in dermatology. Semin Cutan Med Surg..

[77] Esposito S., Garziano M., Rainone V., Trabattoni D., Biasin M., Senatore L., Marchisio P., Rossi M., Principi N., Clerici M. (2015). Immunomodulatory activity of pidotimod administered with standard antibiotic therapy in children hospitalized for community-acquired pneumonia. J. Transl. Med..

[78] Nicolò M., Desideri L.F., Vagge A., Traverso C.E. (2020). Current Pharmacological Treatment Options for Central Serous Chorioretinopathy: A Review. Pharmaceuticals.

[79] Seitz G., Fuchs J., Schaefer J.F., Warmann S.W. (2012). Molecular imaging and photodynamic therapy in hepatoblastoma. Front. Biosci..

[80] Stefini A., Salzer S., Reich G., Horn H., Winkelmann K., Bents H., Rutz U., Frost U., von Boetticher A., Ruhl U. (2017). Cognitive-Behavioral and Psychodynamic Therapy in Female Adolescents with Bulimia Nervosa: A Randomized Controlled Trial. J. Am. Acad. Child. Adolesc. Psychiatry.

